# A retrospective pilot crossover study of protocolized transition from inhaled nitric oxide to inhaled epoprostenol in adult cardiothoracic surgery

**DOI:** 10.1016/j.jhlto.2026.100516

**Published:** 2026-02-13

**Authors:** J.Sam Meyer, Yaron D. Barac, Jhaymie Cappiello, Mary Cooter-Wright, Shanee Dim, Mathew G. Bean, Matthew G. Hartwig, Carmelo A. Milano, Kamrouz Ghadimi

**Affiliations:** a Grey Faculty of Medicine, Tel Aviv University, Tel Aviv, Israel; b Wake Forest Institute for Regenerative Medicine (WFIRM), Wake Forest University School of Medicine, Winston-Salem, NC; c Department of Respiratory Therapy, Boise State University, Boise, ID; d Department of Anesthesiology & Critical Care, Duke University Medical Center, Durham, NC; e Department of Anesthesiology, University of Colorado Health Memorial Hospital, Colorado, Springs, CO; f Department of Surgery, Duke University Medical Center, Durham, NC

**Keywords:** Inhaled epoprostenol, Inhaled nitric oxide, Cardiothoracic surgery, Pulmonary vasodilators, Right ventricular failure, Pulmonary hypertension, Crossover study

## Abstract

**Introduction:**

Inhaled pulmonary vasodilators (iPVDs), including inhaled nitric oxide (iNO) and inhaled epoprostenol (iEPO), are commonly used in the perioperative management of right ventricular dysfunction, pulmonary hypertension, and hypoxemia in cardiothoracic surgery. While randomized trials have shown clinical equivalence between iNO and iEPO, few studies have evaluated protocolized transitions between these agents in heterogeneous postoperative surgical populations. This retrospective pilot study aimed to assess the feasibility, physiologic stability, and oxygenation effects of a unidirectional transition from iNO to iEPO in a real-world cardiothoracic intensive care setting.

**Methods:**

We conducted a retrospective analysis of 77 adult patients who underwent cardiothoracic surgery and were transitioned from iNO to iEPO using a standardized crossover protocol in a single tertiary-care cardiothoracic intensive care unit. Hemodynamic and oxygenation parameters were collected before and after transition. The primary outcomes were changes in mean arterial pressure, pulmonary artery pressures, central venous pressure, cardiac output, mixed venous oxygen saturation (SvO₂), and peripheral oxygen saturation (SpO₂). Paired t-tests were used to compare pre- and post-crossover values.

**Results:**

All patients completed the transition without interruption or adverse clinical events. Hemodynamic parameters remained stable across the transition. Statistically significant reductions were observed in SvO₂ (71.3% to 69.6%, p = 0.0059) and SpO₂ (98.2% to 97.7%, p = 0.0007), though the absolute differences were small. No significant changes were observed in pulmonary artery pressures, MAP, CVP, or cardiac output.

**Conclusions:**

In this pragmatic, retrospective pilot study, protocolized transition from iNO to iEPO was safe, feasible, and well tolerated in a diverse cardiothoracic surgical population. The findings support iEPO as a cost-conscious alternative to iNO in the postoperative setting, with preserved hemodynamic stability and minimal changes in oxygenation. These results contribute to the growing evidence base for implementing standardized iPVD protocols in real-world practice.

## Introduction

Inhaled pulmonary vasodilators (iPVDs), such as inhaled nitric oxide (iNO) and inhaled prostacyclins (e.g., epoprostenol), are frequently used perioperatively in cardiac surgical patients to manage right ventricular (RV) dysfunction, pulmonary hypertension (PH) due to precapillary arteriolar vasoconstriction, and hypoxemia from ventilation-perfusion (V/Q) mismatch. Their use is particularly common in high-risk scenarios such as lung transplantation,^1^, ^2^, ^3^, ^4^, ^5^, ^6^, ^7^, ^8^, ^9^, ^10^, ^11^, ^12^, ^13^, ^14^, ^15^, ^16^, ^17^, ^18^, ^19^, ^20^, ^21^, ^22^, ^23^, ^24^, ^25^ pulmonary thromboendarterectomy for chronic thromboembolic PH,^1^, ^2^, ^3^, ^4^, ^5^, ^6^, ^7^ heart transplantation with RV dysfunction,^13^ and left ventricular assist device (LVAD) implantation.^3^, ^4^ Despite widespread clinical application, these indications remain off-label according to the U.S. Food and Drug Administration.^1^

The 2024 AHA/ACC perioperative guidelines recommend that in patients with precapillary PH undergoing high-risk noncardiac surgery, perioperative use of short-acting iPVDs such as iNO or aerosolized prostacyclins may be reasonable to reduce RV afterload and prevent acute decompensated RV failure (Class IIb recommendation).^14^ The American Heart Association further supports the use of both iNO and inhaled epoprostenol (iEPO) as effective and safe options, typically administered at 20–40 ppm and 10–50 ng/kg/min, respectively.^15^ These agents are favored for their rapid onset and pulmonary selectivity, allowing for targeted vasodilation without the systemic hypotension often seen with intravenous (IV) or oral agents.^4^, ^5^, ^6^, ^7^, ^8^

This pulmonary selectivity stems from localized delivery to ventilated alveoli, causing vasodilation in well-ventilated lung units and improving V/Q matching while reducing pulmonary vascular resistance (PVR).^4^, ^5^, ^6^, ^7^ In contrast, systemic agents such as milrinone, nitroglycerin, sodium nitroprusside, or sildenafil affect both pulmonary and systemic vascular resistance, which can lead to systemic hypotension—an unfavorable effect in patients with RV dysfunction or those undergoing cardiac surgery.^8^, ^9^

Several systematic reviews and meta-analyses support the hemodynamic benefits of iPVDs in the perioperative setting. Inhaled agents have been shown to decrease PVR, increase mean arterial pressure (MAP), and improve right ventricular ejection fraction compared to IV vasodilators.^5^, ^6^, ^7^ Inhaled agents also enhance V/Q matching and oxygenation, whereas IV agents may worsen this by blunting hypoxic pulmonary vasoconstriction.^5^, ^6^, ^7^ However, while some studies suggest reductions in ICU stay and mortality with inhaled prostacyclins, most trials are limited by small sample sizes and low event rates.^5^, ^6^ Milrinone and other inhaled alternatives remain under investigation but lack robust comparative data.^5^

Importantly, randomized trials and meta-analyses comparing iNO and iEPO in cardiac and pulmonary surgical populations have found no significant differences in oxygenation, hemodynamics, length of ICU or hospital stay, duration of mechanical ventilation, or mortality.^8^, ^9^, ^10^, ^11^, ^12^, ^23^ For example, Ghadimi et al. conducted a randomized trial in lung transplant recipients showing clinical equivalence between iNO and iEPO.^23^ These findings have supported broader use of iEPO, especially in centers prioritizing cost-efficiency and operational feasibility.

The American Heart Association and American College of Cardiology emphasize that either agent may be used in the perioperative setting, with the choice often dictated by institutional experience and cost considerations.^14^, ^15^, ^16^ Inhaled epoprostenol is easier to titrate and discontinue due to its short half-life and does not require specialized delivery equipment or monitoring for toxic byproducts like methemoglobin, further supporting its utility.^11^, ^13^

Cost analyses consistently show that iEPO is significantly less expensive than iNO, with reports of sustained cost savings even as overall iPVD use increases.^10^, ^11^, ^12^, ^22^ These savings are often achieved without an increase in bleeding, hypotension, or other adverse events.^10^, ^11^ For instance, Austin et al. reported substantial annual cost reductions after implementing a post-operative iNO-to-iEPO transition protocol in a quaternary care cardiac ICU.^22^

At our institution, historical reliance on iNO and a lack of a standardized delivery protocol initially limited the adoption of iEPO, despite its cost and logistical advantages. In response, we implemented a unidirectional crossover protocol to transition postoperative cardiothoracic patients from iNO to iEPO in the cardiothoracic intensive care unit (CTICU). In this retrospective pilot study, we evaluated the hemodynamic and oxygenation effects of this transition, aiming to assess the feasibility and safety of iEPO as a cost-effective alternative to iNO in the postoperative management of adult cardiothoracic surgical patients.

## Methods

This was a retrospective pilot study evaluating the hemodynamic and oxygenation effects of a standardized, unidirectional crossover protocol for transitioning from inhaled nitric oxide (iNO) to inhaled epoprostenol (iEPO) in adult patients following cardiothoracic surgery. The crossover protocol was implemented as a standardized clinical pathway in the cardiothoracic intensive care unit (CTICU) at Duke University Hospital between July 10, 2015, and December 31, 2016, and this analysis was performed retrospectively on patients who underwent this transition. The study was approved by the institutional review board.

**Figure 1:**
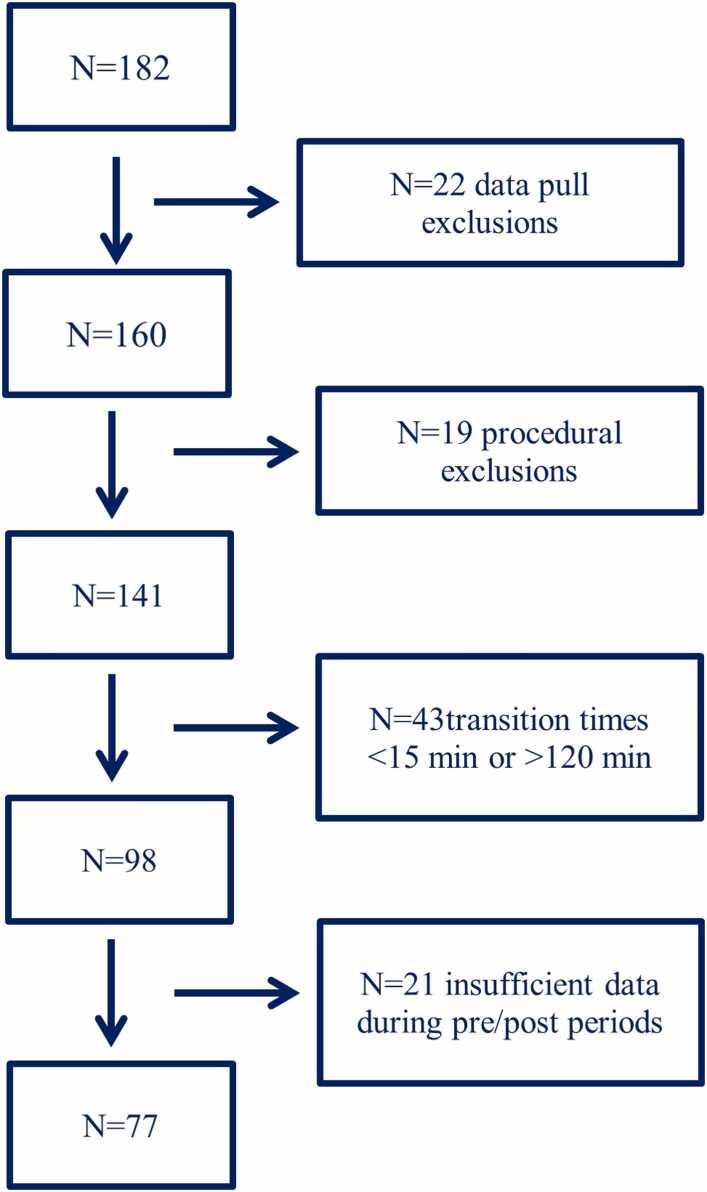
Consort Diagram showing exclusion of patients based on various trial criteria to yield final cohort of 77 patients.

Eligible patients were adults who underwent cardiothoracic surgery and received iNO intraoperatively, followed by protocolized transition to iEPO in the CTICU. Exclusion criteria included patients on veno-venous or veno-arterial extracorporeal membrane oxygenation (ECMO), patients with missing or inconsistent timestamps for initiation or completion of the crossover, and patients with excessively short (<15 min) or prolonged (>120 min) transition periods, which could compromise physiologic comparability. Patients with incomplete documentation of inhaled pulmonary vasodilator (iPVD) therapy or missing outcome data were also excluded. Of 182 screened patients, 77 met inclusion criteria and were included in the final analysis. A patient flow diagram is provided in Figure 1.

All included patients received iNO (20 parts per million) intraoperatively and arrived to the CTICU on continuous iNO. Transition to iEPO was initiated in the CTICU using a vibrating mesh nebulizer system delivering epoprostenol at a fixed dose of 50 ng/kg/min ideal body weight via continuous infusion. During the crossover, iNO was weaned in a stepwise fashion: 20 ppm to 10 ppm, then to 5 ppm, 1 ppm, and finally off, with 10-minute intervals between dose reductions. The duration of crossover ranged between 30 to 60 min, during which the iEPO dose remained constant. All patients included in the analysis completed the full transition protocol without interruption or adverse event requiring return to iNO during the transition itself.

**Figure 2:**
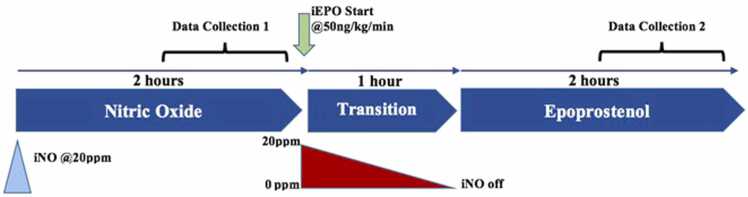
Crossover diagram and Data collection. Data collection time line. All patients arrived to the ICU receiving iNO. Light blue triangle indicates arrival to the ICU with iNO @ 20 ppm. Green arrow indicated the start of the transition period with iEPO @ 50ng/kg/min. Red triangle indicates the gradual weaning of iNO from 20 ppm to off during the transition period. Brackets indicate data collection periods. The first data collection period is over the 60 min prior to the transition period. The second data collection period starts one hour after the transition period ended.

Hemodynamic and oxygenation parameters were extracted from the electronic medical record at two standardized time points: (1) the 60-minute period immediately prior to the initiation of crossover, and (2) the second hour following complete discontinuation of iNO, which allowed for a 1-hour washout period post-transition. Variables collected included mean arterial pressure (MAP), systolic, diastolic, and mean pulmonary artery pressures (PAPs, PAPd, PAPm), central venous pressure (CVP), cardiac output (CO), mixed venous oxygen saturation (SvO₂), and peripheral oxygen saturation (SpO₂). Ventilator parameters such as fraction of inspired oxygen (FiO₂), positive end-expiratory pressure (PEEP), and arterial oxygen tension (PaO₂) were not consistently available for analysis and are not reported in this study. This limitation is discussed further in the Discussion section. A data collection timeline is provided in Figure 2.

The primary outcomes were changes in hemodynamic and oxygenation parameters before and after the transition from iNO to iEPO. All patients served as their own control in this crossover design. Any subsequent re-initiation of iNO after completion of the crossover was not part of the standardized protocol and occurred at clinician discretion later in the ICU course. Descriptive statistics were used to summarize baseline characteristics. Continuous variables were reported as means with standard deviations. Paired t-tests were used to compare pre- and post-transition values for each physiologic parameter. Due to the retrospective nature of the study and absence of raw-level data, no additional statistical modeling or normality testing was performed, and no imputation was applied for missing data. A two-sided p-value of <0.05 was considered statistically significant. All analyses were performed using SAS version 9.4 (SAS Institute, Cary, NC).

## Results

A total of 77 adult cardiothoracic surgery patients met study criteria and were included in the final analysis. The mean age was 58.2 ± 12.9 years, and 61.0% were male. The most common surgical procedure was ventricular assist device (VAD) implantation (36 patients, 46.8%), followed by heart transplantation (14 patients, 18.2%), lung transplantation (14 patients, 18.2%), and other cardiothoracic procedures (13 patients, 16.9%) including coronary artery bypass grafting (CABG), valve, and pulmonary thromboendarterectomy. Right ventricular dysfunction was present in 74.0% of patients, and 39.0% had documented pulmonary hypertension. Baseline patient characteristics and intraoperative inotrope use are presented in Table 1.

**Table 1:** Patient Characteristics

Number of Patients	N=77
Age (years)	58.2 (12.9)
Gender(Male)	47 (61.0%)
Comorbidities	
Right Heart Dysfunction	57 (74.0%)
CAD	39 (50.6%)
Renal Failure	28 (36.4%)
PAH	30 (39.0%)
DM	22 (28.6%)
HTN	22 (28.6%)
Obesity	17 (22.1%)
OSA	16 (20.8%)
COPD	18 (23.4%)
AMI	13 (16.9%)
Cancer	17 (22.1%)
Idiopathic Pulmonary Fibrosis	11 (14.3%)
Liver Disease	4 (5.2%)
Asthma	3 (3.9%)
CF	3 (3.9%)
Peripheral Vascular Disease	2 (2.6%)
Primary Surgery Type	
Heart Transplant	14 (18.2%)
Lung Transplant	14 (18.2%)
LVAD	36 (46.8%)
Valve	9 (11.7%)
Other	4 (5.2%)
Time from end of surgery to pre-cross over evaluation start (hrs)	17.1 (20.9)
Inotropes during iNO period	
Epinephrine	72 (93.5%)
Norepinephrine	11 (14.3%)
Milrinone	28 (36.4%)
Dopamine	22 (28.6%)
Dobutamine	3 (3.9%)
Vasopressin	31 (40.3%)
iNO to iEPO crossover time (min)	56.0 (26.0)
Inotropes during iEPO period	
Epinephrine	69 (89.6%)
Norepinephrine	7 (9.1%)
Milrinone	27 (35.1%)
Dopamine	23 (29.9%)
Dobutamine	3 (3.9%)
Vasopressin	27 (35.1%)

Age is described with the mean (SD). The other variables are summarized using frequency (%).

Abbreviations: VAD = ventricular assist device; CABG = coronary artery bypass grafting; OSA = obstructive sleep apnea; COPD = chronic obstructive pulmonary disease; AMI = acute myocardial infarction; DM = diabetes mellitus; HTN = hypertension; CAD = coronary artery disease; PAH = pulmonary arterial hypertension; CF = cystic fibrosis; BOLT = bilateral orthotopic lung transplant; SOLT = single orthotopic lung transplant; iNO = inhaled nitric oxide; iEPO = inhaled epoprostenol; CTICU = cardiothoracic intensive care unit.

All patients underwent protocolized transition from iNO to iEPO in the CTICU, with a mean crossover time of 56.0 ± 26.0 min. No protocol deviations or interruptions were recorded. No patient required re-initiation of iNO during the crossover period. Hemodynamic and oxygenation data were available for all patients during both the pre- and post-transition periods.

Mean arterial pressure was 79.0 ± 9.8 mm Hg during iNO use and 78.8 ± 9.2 mm Hg during iEPO use (p = 0.85). Mean pulmonary artery pressure was 24.1 ± 6.4 mm Hg during iNO and 23.7 ± 6.1 mm Hg during iEPO (p = 0.35). Systolic and diastolic pulmonary artery pressures, CVP, and cardiac output were also not significantly different between iNO and iEPO administration (Table 2). Cardiac output increased from 4.9 ± 1.1 L/min to 5.1 ± 1.4 L/min after transition (p = 0.058).

**Table 2:** Hemodynamic and Oxygenation Parameters Pre/Post Transition with Respective *p* Values

	iNO (n=77)	iPGI_2_ (n=77)	Pre-Post Change	Univariate p-value	Multivariate p-value
MAP (mm Hg)	79.0 (9.8)	78.8 (9.2)	-0.18 (8.37)	0.8475	0.8475
PAPm (mm Hg)	24.1 (6.4)	23.7 (6.1)	-0.35 (3.32)	0.3511	0.3155
MAP:PAPm	3.5 (0.9)	3.5 (1.0)	0.06 (0.54)	0.3516	0.3516
PAPs (mm Hg)	35.0 (10.4)	34.6 (9.7)	-0.41 (5.23)	0.4838	0.4838
PAPd (mm Hg)	17.6 (5.2)	17.5 (5.0)	-0.15 (3.42)	0.7074	0.7074
CVP (mm Hg)	12.0 (8.0)	11.1 (4.8)	-0.75 (8.16)	0.4313	0.3506
CO	4.9 (1.1)	5.1 (1.4)	0.24 (1.08)	0.0578	0.0578
SvO2 (%)	71.3 (8.3)	69.6 (8.7)	-1.73 (5.42)	**0.0059**	**0.0059**
SpO2 (%)	98.2 (2.0)	97.7 (2.3)	-0.53 (1.34)	**0.0007**	**0.0007**

Data are presented as mean ± standard deviation. FiO₂, PEEP, and PaO₂ were not consistently available and are not included. Abbreviations: MAP = mean arterial pressure; PAPm = mean pulmonary artery pressure; PAPs = systolic pulmonary artery pressure; PAPd = diastolic pulmonary artery pressure; CVP = central venous pressure; CO = cardiac output; SvO₂ = mixed venous oxygen saturation; SpO₂ = peripheral oxygen saturation; iNO = inhaled nitric oxide; iEPO = inhaled epoprostenol.

**Figure 3:**
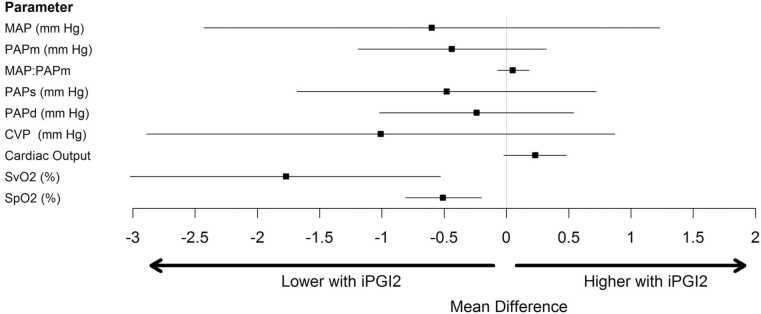
Mean differences following transition to iEPO.

Mixed venous oxygen saturation (SvO₂) decreased from 71.3% ± 8.3% during iNO to 69.6% ± 8.7% during iEPO (p = 0.0059). Peripheral oxygen saturation (SpO₂) decreased from 98.2% ± 2.0% to 97.7% ± 2.3% (p = 0.0007). All other oxygenation and hemodynamic variables are reported in Table 2. A graphical summary of mean differences before and after transition is provided in Figure 3.

## Discussion

This retrospective pilot study evaluated a standardized crossover protocol for transitioning from inhaled nitric oxide (iNO) to inhaled epoprostenol (iEPO) in a diverse cohort of adult patients recovering from cardiothoracic surgery. To our knowledge, this represents the largest real-world crossover cohort published to date in this population. Across 77 patients, including individuals undergoing ventricular assist device implantation, heart transplantation, and lung transplantation, we observed stable hemodynamic parameters during the transition, with only small changes in oxygenation metrics.

Consistent with prior literature, we found no significant changes in pulmonary artery pressures, central venous pressure, mean arterial pressure, or cardiac output before and after transitioning to iEPO. Mixed venous oxygen saturation (SvO₂) and peripheral oxygen saturation (SpO₂) decreased by 1.7% and 0.5%, respectively, with statistical significance. However, these differences are modest in absolute terms and occurred in the absence of hemodynamic instability or adverse events. The small magnitude of these changes, combined with the retrospective design and the absence of key ventilatory parameters (e.g., FiO₂, PEEP, PaO₂), limits the ability to assess their clinical significance. Nonetheless, all patients completed the crossover protocol without interruption, and no patient required a return to iNO during the transition phase.

These findings align with the growing body of evidence supporting the clinical equivalence of iNO and iEPO. The American Heart Association has issued a scientific statement recognizing both agents as reasonable options for perioperative pulmonary hypertension and right ventricular dysfunction, citing comparable efficacy and safety profiles.^15^ Large randomized trials and meta-analyses have similarly demonstrated no significant differences in key clinical outcomes between iNO and iEPO, including pulmonary artery pressures, cardiac output, duration of mechanical ventilation, ICU and hospital length of stay, or mortality.^11^, ^19^, ^20^, ^23^, ^24^ The largest randomized controlled trial to date in lung transplantation, conducted by Ghadimi et al., found no difference in rates of primary graft dysfunction or secondary outcomes between iEPO and iNO²³. Other studies have noted potential mortality benefit for inhaled prostacyclins, but this finding remains inconclusive due to low event rates and limited sample sizes.^20^, ^24^, ^25^

In our study, protocolized transition from iNO to iEPO was successfully implemented in a mixed, high-acuity surgical population with no reported adverse events. This supports the feasibility of adopting inhaled epoprostenol protocols even in resource-intensive settings such as heart and lung transplantation or mechanical circulatory support. Recent consensus documents and perioperative guidelines also emphasize institutional context in selecting iPVD agents, acknowledging both as acceptable first-line options depending on resource availability and provider familiarity.^14^, ^26^

Cost-effectiveness remains a major driver in the decision to adopt iEPO over iNO. Inhaled epoprostenol has been shown to be significantly less expensive than iNO, both in acquisition costs and equipment requirements, and does not necessitate proprietary delivery systems or methemoglobin monitoring.^11^, ^21^, ^22^ Austin et al. estimated an annual cost savings of over $1 million after implementing an iNO-to-iEPO transition protocol in a large quaternary care ICU.^22^ Real-world data continue to support these economic advantages without compromising safety or clinical outcomes.^21^, ^24^, ^27^

## Limitations

This study has several important limitations. First, it was a single-center, retrospective analysis of a standardized clinical protocol, and thus subject to inherent limitations in data completeness and potential for selection bias. Although the crossover design allowed each patient to serve as their own control, the direction of transition—from iNO to iEPO—was unidirectional and not randomized. As such, we cannot exclude the possibility of time-related confounding or order effects. In addition, because this was a pragmatic implementation study, data on ventilator settings—including fraction of inspired oxygen (FiO₂), positive end-expiratory pressure (PEEP), and arterial partial pressure of oxygen (PaO₂)—were not consistently available and could not be analyzed. This limits the interpretability of the statistically significant but small changes in SvO₂ and SpO₂ observed during the crossover. Furthermore, the study did not quantify or evaluate the initial physiologic response to either iNO or iEPO independently, but instead focused solely on stability across the transition. Approximately one-third of patients were later re-initiated on iNO after completion of the crossover; none required iNO re-initiation during the crossover itself. Because re-initiation was not protocolized and indications were not systematically captured, these events likely reflect clinician-directed responses to evolving postoperative physiology rather than crossover protocol failure. Finally, although this represents one of the largest crossover cohorts in this field, the sample size was not powered to detect rare adverse events or outcomes across specific subgroups, such as transplant recipients or mechanical circulatory support patients.

## Conclusions

In this retrospective pilot study of adult cardiothoracic surgical patients, we found that protocolized transition from inhaled nitric oxide to inhaled epoprostenol was feasible and well tolerated, with preserved hemodynamic stability and only small, statistically significant changes in oxygenation. No patients required discontinuation of the transition protocol or re-initiation of iNO due to adverse clinical events during the crossover period. These findings add to a growing body of evidence—supported by randomized trials, meta-analyses, and societal guidelines—demonstrating clinical equivalence between iNO and iEPO in the perioperative setting. Given its lower cost, simplified delivery, and similar safety profile, inhaled epoprostenol represents a viable and cost-conscious alternative to iNO for institutions seeking to standardize pulmonary vasodilator therapy following cardiothoracic surgery. Future prospective studies are warranted to determine comparative effectiveness in specific high-risk subgroups and to optimize iPVD delivery strategies across different perioperative settings.

## Funding Sources and Relevant Disclosures

The authors have no funding sources nor relevant disclosures to declare. Yaron D. Barac declares no financial support in the production of this manuscript.

## Declaration of Competing Interest

The authors declare that they have no known competing financial interests or personal relationships that could have appeared to influence the work reported in this paper.
